# Development and Validation of the Robust - Pandemic Coping Scale (R-PCS)

**DOI:** 10.3389/fpsyg.2021.725344

**Published:** 2021-09-09

**Authors:** Roberto Burro, Giada Vicentini, Emmanuela Rocca, Veronica Barnaba, Rob Hall, Daniela Raccanello

**Affiliations:** ^1^Department of Human Sciences, University of Verona, Verona, Italy; ^2^Environmetrics Pty Ltd., Killara, NSW, Australia; ^3^Department of Psychology, Macquarie University, Sydney, NSW, Australia

**Keywords:** measures, coping strategies, pandemic, scale development, factor analysis, invariance analysis, Rasch model

## Abstract

The psychological consequences of epidemics/pandemics, such as the COVID-19 pandemic, include an increase in psychopathological symptoms, such as depression, anxiety, and stress, and negative emotions, such as fear. However, relatively little attention has been paid to how people cope with the pandemic. Coping is a multi-component process, helping to diminish the traumatic impact of stressful events in a variety of ways. We studied how university students coped with the first wave of the COVID-19 pandemic, by developing the Robust - Pandemic Coping Scale (R-PCS), a new scale for measuring coping strategies related to epidemics/pandemics. The scale is based on a classification of coping strategies referred to the needs of competence, relatedness, and autonomy. To create a robust scale, such that the item values would be independent of the sample used for developing it, we employed Rasch modeling. We used a sample of 2,987 Italian university students who participated in an online survey including the R-PCS and the Power to Live with Disasters Questionnaire (PLDQ), during March 2020. First, we applied a dual approach combining exploratory and confirmatory factor analyses, which supported the goodness of a 4-factor model (i.e., Despair, Adjustment, Proactivity, and Aversion) for the R-PCS, invariant across gender and age of respondents (younger or as old as 23 years, older than 23 years). We then transformed the raw scores of the R-PCS into interval logit scale scores applying the Rasch model. Second, our findings supported the discriminant validity and the criterion validity of the R-PCS, examining the correlations with the PLDQ. They also confirmed its predictive validity: the R-PCS scores were related to 2-month-later enjoyment and anger, indicating that Adjustment and Proactivity were adaptive while Despair and Aversion were maladaptive. Third, our study revealed gender and age differences: the scores were higher for Despair, Adjustment, and Proactivity for females; for Aversion for males; and for Proactivity for students older than 23 years. The study suffers from limitations related to social desirability, gender imbalance, and self-selection effects in the recruitment.

## Introduction

The outbreak of the COVID-19 pandemic posed a new and unexpected challenge worldwide, given the lack of readily available treatments or well-established solutions. The spread of the virus across countries and continents was marked by uncertainty and unpredictability (Vinkers et al., 2020), factors that are linked to increases in people’s anxiety (Estes and Thompson, 2020). During the pandemic, policies for limiting the spread of the virus tended to be in a state of flux and were adjusted in response to new data and/or political pressure. The search for an effective vaccine was an ongoing priority. Because of the general uncertainty, there were major changes in the patterns of personal, social, cultural, and economic activities that had previously defined as “normal life.” This was particularly so for university students given that they suffered very early and more extensively from the constraints aimed at preventing the spread of the virus. In many cases, they were forced to live within their dwellings or at some distance from family or friends; they had to respect social distancing and give up on-site university life and abruptly adapt to e-learning modalities. They were especially susceptible to experiencing economic uncertainty about both their current studies and their future profession. Given the key role of coping strategies for limiting and mitigating the negative consequences of disasters including pandemics, it is important to be able to separate the strategies that are most effective from those that are not so that people can be given the most effective psychological advice. To this end, we developed a self-report scale assessing a variety of pandemic-related coping strategies, using a dual approach combining the strengths of factor analyses and the Rasch model (Bond and Fox, 2007), i.e., the Robust - Pandemic Coping Scale (R-PCS).

### Psychological Functioning, Coping Strategies, and Pandemics

Several studies, with samples ranging from younger to older adults, have shown that the COVID-19 pandemic has psychopathological consequences including anxiety, depression, panic behaviors, emotional distress, and various symptomatic responses (Arslan et al., 2020; Fitzpatrick et al., 2020; Gallagher et al., 2020; García-Portilla et al., 2020; Nicomedes and Avila, 2020; Rodríguez-Rey et al., 2020; Dozois and Mental Health Research Canada, 2021). Notably, it has been observed that the level of anxiety and fear was higher in the regions with higher COVID-19 rates (Fitzpatrick et al., 2020). Some authors have validated specific scales to assess fear and anxiety, such as the Fear of COVID-19 Scale (Ahorsu et al., 2020), which is a unidimensional scale based on seven items. This scale has already been employed in Bangladesh, Iran, Israel, Italy, Japan, New Zealand, Russia and Belarus, Saudi Arabia, Turkey, and Vietnam (Alyami et al., 2020; Bitan et al., 2020; Haktanir et al., 2020; Reznik et al., 2020; Sakib et al., 2020; Soraci et al., 2020; Wakashima et al., 2020; Winter et al., 2020). Using this scale, Satici et al. (2020) found that fear mediated the relation between intolerance of uncertainty and mental well-being: People who were more intolerant of uncertainties were also more afraid, and, in turn, their mental health was worse. Lee (2020) developed a 5-item scale using a sample of North American adults to assess anxiety – the Coronavirus and Anxiety Scale. This scale distinguishes between cognitive, behavioral, and psychological disturbances. Fear and anxiety can be considered as emotional reactions, which can, in fact, facilitate adjustment (Porcelli, 2020); however, extreme levels of fear can result in highly dysfunctional reactions and behaviors (Cheng and Tang, 2004). Some studies focused on university students and revealed increases in anxiety, depression, stress, post-traumatic stress disorder, perceptions of loneliness, fear, and worry (Cao et al., 2020; Elmer et al., 2020; Husky et al., 2020; Odriozola-González et al., 2020; Son et al., 2020; Tang et al., 2020).

Some of the negative consequences of pandemics can be mitigated by coping strategies. Coping is a multi-dimensional process for facing stressful situations (Lazarus and Folkman, 1984; Skinner et al., 2003; Skinner and Zimmer-Gembeck, 2007). According to Lazarus and Folkman’s transactional model, there are three stages of evaluation that help individuals to adjust to threatening external situations. The first stage involves assessing the likely extent of damage or loss that might be incurred; the second stage involves identifying relevant coping strategies based on the individual and social resources that are available to the individual; and the third stage involves estimating the efficacy of each of these strategies. Coping strategies are commonly characterized as fitting into a typology based on the extent to which they focus on problems or emotions (Lazarus and Folkman, 1984) and the “fight or flight” responses they elicit (Schaefer and Moos, 1992). In the literature, there are several taxonomies classifying coping. In the attempt to propose a developmental classification including a wide range of coping strategies, Zimmer-Gembeck and Skinner (2011) elaborated a taxonomy incorporating three categories focused on three basic human needs, i.e., the needs for competence, relatedness, and autonomy (Deci and Ryan, 1985), that can be challenged or threatened by uncertain events. For each category, there are two adaptive and two maladaptive families of coping strategies that are typically activated in the face of perceived challenges or threats. Concerning the need for competence, the adaptive responses include problem solving (i.e., focusing on the problems with an analytical approach) and information seeking (i.e., searching for information by oneself or with others) while the maladaptive responses include helplessness (i.e., adopting a helpless or confused attitude when faced with situational demands) and escape (i.e., attempting to avoid the problem behaviorally or psychologically). In respect to the need for relatedness, the taxonomy includes self-reliance (i.e., focusing on emotional expression, understanding, or regulation) and support seeking (i.e., searching for concrete or psychological social support) in contrast to delegation (i.e., feeling of being out of control) and social isolation (i.e., withdrawing or refusing social contact). As for the need for autonomy, the taxonomy includes accommodation (i.e., attempting to adjust actively or by cognitively restructuring the situation) and negotiation (i.e., trying to increase the available options by contracting, persuading, or identifying priorities) in contrast with submission (i.e., adopting a passive attitude with intrusive thoughts or rumination) and opposition (i.e., demonstrating a marked refusal to cooperate). In Table 1, we report some examples of studies of coping strategies that can be coded according to this classification.

**Table 1:** Examples of coping strategies from previous studies and dimensions of the Robust - Pandemic Coping Scale (R-PCS) relative to each needs-based category.

	Need for competence		Need for relatedness		Need for autonomy	
	Examples of studies	Dimensions of the R-PCS	Examples of studies	Dimensions of the R-PCS	Examples of studies	Dimensions of the R-PCS
Challenges	Fullana et al., 2020: having a healthy/balanced diet, not reading COVID-19-related news very often. Park et al., 2021: active coping. Shamblaw et al., 2021: active coping, planning, informational support. Wakashima et al., 2020: protective behaviors, stockpiling supplies, monitoring health. Waselewski et al., 2020: prevention behaviors	Proactivity	Cao et al., 2020: social support. Park et al., 2021: regulation of emotions, social support. Shamblaw et al., 2021: emotional support. Son et al., 2020: support seeking. Waselewski et al., 2020: staying connected to people	Adjustment	Fullana et al., 2020: following a routine, cultivating hobbies, staying outdoors. Shamblaw et al., 2021: positive reframing, acceptance. Park et al., 2021: distraction. Son et al., 2020: sleeping longer, distracting by doing other tasks, meditation and breathing exercises, spiritual measures, keeping to routines, positive reframing. Waselewski et al., 2020: relaxing, thinking positively, keeping busy, following routines, cultivating hobbies, studying, having physical exercise	Adjustment
Threats	Shamblaw et al., 2021: avoidant coping. Son et al., 2020: ignoring the news	Despair	Elmer et al., 2020: isolation from social networks, lack of interaction, lack of emotional support, physical isolation. Panayiotou et al., 2021: difficulties in expressing and verbalizing emotions	Despair	Bakker and van Wingerden, 2021: rumination. Satici et al., 2020: rumination. Shamblaw et al., 2021: rumination. Son et al., 2020: drinking, smoking	Despair Aversion

Some studies have recently examined how adults coped during the pandemics, exploring the links between coping strategies and some indicators of positive psychological functioning and/or psychopathological symptoms (Fullana et al., 2020; Wakashima et al., 2020; Bakker and van Wingerden, 2021; Park et al., 2021; Shamblaw et al., 2021). Shamblaw et al. (2021) explored adaptive strategies in a sample of American adults and found that a variety of strategies, such as active coping, positive reframing, planning, acceptance, emotional support, and the use of informational support was associated with lower levels of depression and higher levels of quality of life. Of the strategies they examined, they found that the most beneficial was positive reframing. In another study involving Americans, skills for regulating emotions, active coping, and distraction were significant predictors of a lower level of distress; while social support seemed less effective (Park et al., 2021). Other studies have focused on correlates of behavioral coping. Wakashima et al. (2020) found, among a Japanese sample, that the level of COVID-19-related fear was positively linked to protective behaviors (implying adherence to safety measures), stockpiling supplies, and monitoring one’s health. Fullana et al. (2020) found that among a sample of Spanish adults, behavioral coping, such as having a healthy/balanced diet and not reading news/updates about COVID-19 very often was negatively associated with symptoms like anxiety and depression; in addition, following a routine, cultivating hobbies, and staying outdoors or looking outside were negatively linked to depression. All the strategies examined by these authors can be classified as challenge-related coping strategies focused on the need for competence, relatedness, and/or autonomy according to Zimmer-Gembeck and Skinner’s (2011) classification.

Some data also suggest that most of the maladaptive coping strategies classified by Zimmer-Gembeck and Skinner (2011) for dealing with threats are, in fact, detrimental. In the research by Shamblaw et al. (2021), using avoidant coping (including strategies such as denial, substance use, venting, behavioral disengagement, distraction, and self-blame) was related to increases in depression and anxiety and decreases in quality of life. In a sample of Dutch adults, rumination was associated with lower well-being, operationalized in terms of depression, exhaustion, and less vigor (Bakker and van Wingerden, 2021). Research using a sample of Turkish participants found that rumination mediated the relation between intolerance of uncertainty and mental well-being, i.e., more intolerant people ruminated more, and in turn people who ruminated more had lower indicators of mental health; moreover, rumination was associated with increases in fear (Satici et al., 2020).

While most studies have used adult samples from a range of population groups, relatively little research has focused specifically on how university students coped with the COVID-19 pandemic (for exceptions, see Son et al., 2020; Waselewski et al., 2020; Panayiotou et al., 2021). Among Cypriot university students, difficulties in expressing and verbalizing emotions on the one hand and difficulties in having access to a repertoire of emotion regulation strategies on the other hand predicted the decrease in quality of life due to the outbreak of the pandemic (Panayiotou et al., 2021). Waselewski et al. (2020) used a qualitative approach to explore how 14-to-24-year olds coped with the pandemic. Using content analysis, they identified a variety of strategies such as following prevention behaviors, staying connected to people, relaxing and thinking positively, keeping busy, following routines, cultivating hobbies, studying, or having physical exercise. American university students adopted support seeking and other strategies like ignoring the news, sleeping longer, distracting themselves by doing other tasks, drinking, or smoking, meditation and breathing exercises, spiritual measures, keeping to routines, and positive reframing strategies to cope with stress and anxiety due to COVID-19 (Son et al., 2020). Research among Swiss university students found that isolation from social networks, lack of interaction and emotional support, and physical isolation were associated with negative mental health (Elmer et al., 2020), while for Chinese university students, social support was negatively correlated with anxiety (Cao et al., 2020). However, notwithstanding the interest in coping strategies, there are, to our knowledge, no instruments specifically designed for use among university students to assess a wide range of coping strategies related to pandemics and/or epidemics.

### Measurement of Disaster-Related Coping Strategies

Coping strategies are typically measured through self-report instruments, which have both disadvantages and advantages (Pekrun and Bühner, 2014). One limitation is that they only capture conscious psychological processes (although, coping strategies are usually conscious; Lazarus and Folkman, 1984; Compas et al., 2001). They may also be prone to desirability biases. However, being relatively cheap to implement and easy to be adapted to many different contexts, self-report instruments are still the most commonly used tools for accessing individuals’ inner worlds.

Most of the published studies for assessing coping strategies related to COVID-19 used self-report questionnaires (e.g., Fullana et al., 2020; Wakashima et al., 2020; Bakker and van Wingerden, 2021; Park et al., 2021; Shamblaw et al., 2021) and only a few utilized open-ended questions with content analysis (e.g., Son et al., 2020; Waselewski et al., 2020). Generally, researchers who studied disaster-related coping strategies did not develop specific measures focused on disasters. As an exception, some authors validated the Power to Live with Disasters Questionnaire (PLDQ), a questionnaire measuring personality characteristics useful for coping with disasters, both in long (Sugiura et al., 2015) and short versions (Ishibashi et al., 2019). However, the PLDQ is not focused on disaster-related coping strategies. Developing a scale with pandemic-specific items was a response to the need for measuring coping strategies with an instrument relevant to the specific characteristics of this disaster. Pandemics and epidemics are typically characterized by a very long emergency phase, unlike, for example, earthquakes or tornados. During this phase, psychological interventions are urgently needed to assist people employ appropriate coping strategies. It follows, that it is important to have an instrument from which evidence-based recommendations can be made.

Despite the existence of some specific questionnaires to assess COVID-19-fear and anxiety (Ahorsu et al., 2020; Lee, 2020), to our knowledge, there is a lack of measures concerning coping strategies. Therefore, there was a need to develop a robust valid instrument focused on disaster-related coping strategies in general and on pandemic-related coping strategies in particular. By “robust and valid,” we mean an instrument that meets the conditions of fundamental measurement, i.e., obtaining measurements not built from a foundation of other measurements, and which follows an additive logic (Bond and Fox, 2007). Fundamental measurement typically characterizes measurements made using basic units in the physical and natural sciences, while it is not so frequently encountered in the social sciences. To reach this objective, we used Rasch modelling (Rasch, 1960; Andrich, 1988) as the second stage of a two-stage approach to developing the scale in which exploratory factor analysis (EFA) and confirmatory factor analysis (CFA) were used to identify an initial set of items and then the items were fit to the Rasch model. Using such approach is particularly welcomed when the aim of the researchers is to diminish a large set of items to identify a lower number of scale scores (Christensen, 2021). In the literature, such approach has been amply used (e.g., Vidotto et al., 2010; Panella et al., 2012; Chiu et al., 2020). A major benefit of using Rasch scaling is that, when data can be shown to fit the Rasch model, it is possible to obtain for each item a score that is independent of characteristics of the sample of respondents and items, i.e., obtaining sample-free and test-free measures. These measures form an interval logit scale (Burro, 2016). There are several ordered steps that must be followed to apply the Rasch model and to verify its goodness of fit. First, one must evaluate the construct validity of the scale (Bretagnolle, 2002; Kang et al., 2018), the local independence (Marais, 2013; Debelak and Koller, 2020), the unidimensionality (Christensen et al., 2002; Smith, 2002), and the absence of differential item functioning (DIF; Tennant et al., 2004; Hagquist and Andrich, 2017), i.e., see that the instrument functions in the same way across different groups of participants. Second, one can use indices, such as the person separation index (PSI) or Cronbach’s alpha (Wright, 2001; Kreiner and Christensen, 2013), to establish the reliability of the scale. Finally, one looks at the level of correspondence between the distribution of the calibrated items and that of the participants (Wright and Masters, 1982).

In some cases, it is possible that one or more of the assumptions concerning internal construct validity are not met. When this happens, one can implement an iterative procedure using different modification strategies (Linacre, 2002; Tennant and Conaghan, 2007), to account for violations of monotonicity (e.g., item rescoring), for violations of local independence (e.g., item grouping or “testlets” creation), and for the presence of DIF (e.g., item splitting). If the previous strategies do not work, one can delete critical items, and repeat the steps in the process. When all the assumptions are satisfied, the final step is to verify the fit of the model.

### Aims

This study aimed at developing and testing the psychometric properties of a new scale, the R-PCS, designed to measure a range of coping strategies related to epidemics and pandemics. The scale was inspired by the classification proposed by Zimmer-Gembeck and Skinner (2011) that incorporates adaptive and maladaptive coping strategies pertaining to three functions, i.e., competence, relatedness, and autonomy.

The questionnaire contained three items for each of the 12 families of coping strategies proposed by Zimmer-Gembeck and Skinner (2011). We expected to identify different dimensions pertaining to categories reflecting adaptive or maladaptive coping strategies, that is, we expected to find at least one dimension focused on challenges and at least one dimension focused on threats. We also anticipated finding further dimensions linked to specific coping strategies (Hypothesis 1a). Moreover, we hypothesized that the factorial structure of the scale was invariant across gender and age of respondents (Hypothesis 1b). Then, we transformed each identified dimension applying the Rasch analysis.

The second aim was to study the validity of the R-PCS. Concerning the discriminant validity, we expected its dimensions to be independent (Hypothesis 2a). As regards the criterion validity, we expected that the dimensions would correlate with the factors of a scale designed to measure personality characteristics useful for coping with disasters, the PLDQ (Hypothesis 2b; Ishibashi et al., 2019). Pertaining to the predictive validity, we expected that the dimensions reflecting adaptive coping strategies would be positively related to enjoyment and negatively related to anger (Raccanello et al., 2021b) and vice versa for the dimensions reflecting maladaptive coping strategies (Hypothesis 2c) measured 2 months after the administration of the R-PCS.

The third aim was to examine interindividual differences in the R-PCS, examining possible differences related to gender and age of respondents.

## Materials and Methods

### Participants

The sample consisted of 2,987 university students (*M*_age_=25.51years, *SD*=6.62; 79% females), from the University of Verona in Northern Italy. The participants were attending bachelor’s degree courses (58%), master’s degree courses (36%), or PhD and other specialization courses (6%). The sample was divided into two groups by splitting the total sample at the median age (24 years), with one group of 1,476 students (49% of the sample) being 23 years of age or younger and the other group of 1,511 students (51% of the sample) being older than 23 years. Concerning their health status as related to the 2020 COVID-19 pandemic at the time of the survey, 0.36% of them had been tested for novel coronavirus and had resulted positive, 1.43% had been tested for novel coronavirus and had resulted negative, and 98.21% had not been tested.

### Procedure

The study was approved by the Director of the Head Office General Management of the University of Verona and by the Ethical Committee of the Department of Human Sciences of the same university (protocol n. 118846/2020). We sent an email to all the students attending the University of Verona during the academic year 2019–2020 (more than 25,000 students), inviting them to participate in an online survey on COVID-19 and emotions. The students gave their informed consent before participating. This work is part of a longitudinal study, in which we have administered the survey every 2 months since the outbreak of the pandemic. The first administration of the Italian language survey was between March 23 and April 1, 2020. In this paper, we also report data from a sub-sample of 998 students who participated in the second administration of the survey between May 18 and May 24, 2020.

#### Measures

##### Robust - Pandemic Coping Scale

We developed the R-PCS as follows. We conducted an extensive review of the literature on coping strategies used to deal with natural disasters. Two studies were particularly important in informing the development of the scale. One was a meta-analysis of relevant studies involving children and adolescents (Raccanello et al., 2019) and the other, a study in which adults reported adaptive strategies used to cope with earthquakes (Raccanello et al., 2021a). The literature review was followed by a process, in which four experts in developmental and educational psychology independently created a set of adaptive and maladaptive strategies that could be used to cope with the negative psychological consequences of pandemics, basing their work on previous research (e.g., Raccanello et al., 2019, 2020a,b, 2021a; Vicentini et al., 2020). A panel of judges (consisting of the four previously mentioned experts and two other experts in general psychology and education) discussed the set of items and retained 36 of the initial pool. These 36 items included adaptive or maladaptive coping strategies as identified by Zimmer-Gembeck and Skinner (2011). Some examples of the coping strategies to which the 36 items referred were as follows: adaptive strategies related to competence included problem solving, e.g., *Behaving in safe ways (for example washing my hands frequently)*, and information seeking/giving, e.g., *Looking for information from reliable sources* while among maladaptive strategies there was helplessness, e.g., *Thinking that nobody can help me*, and escape, e.g., *Pretending that there is no emergency*. Among the strategies focused on relatedness, adaptive strategies comprised, for example, self-reliance, e.g., *Keeping calm*, and support seeking/giving, e.g., *Collaborating with others* while among maladaptive strategies there was delegation, e.g., *Panicking*, and social isolation, e.g., *Being selfish*. Finally, adaptive coping strategies focused on autonomy included accommodation, e.g., *Keeping myself busy (for example playing or studying)*, and negotiation, e.g., *Creating new routines if usual ones cannot be followed*. Maladaptive strategies included submission, e.g., *Thinking that safety measures are not useful*, and opposition, e.g., *Thinking that media and politicians are exaggerating the situation*.

Each item was rated on a 5-point scale (1=*never* and 5=*always*) to indicate the frequency with which that strategy was used (*Think to how you have coped with emotions such as fear, sadness, and anger, that you could have felt since the outbreak of the pandemic. Please indicate how frequently you have used the following strategies*). In Table 2, we list the 20 items that were retained for the final version of the R-PCS after the statistical analyses.

**Table 2:** Item description in the English and Italian versions and factor loadings for the four factors.

Factor name	Item number	Italian version	English translation	Loadings Factor 1	Loadings Factor 2	Loadings Factor 3	Loadings Factor 4
Despair	6	Farsi prendere dal panico	Panicking	**0.761**	0.065	−0.111	−0.146
	27 (reversed)	Mantenere la calma	Keeping calm	**0.705**	0.024	−0.288	−0.185
	7	Pensare solo all’emergenza	Overthinking about the emergency	**0.558**	−0.100	0.115	−0.057
	32	Pensare che la situazione non migliorerà mai	Thinking that things will never get better	**0.505**	−0.013	0.114	0.167
	10	Pensare che nessuno possa aiutarci	Thinking that nobody can help me	**0.497**	−0.093	0.107	0.162
Adjustment	26	Inventarsi routine nuove se non si possono seguire quelle abituali	Creating new routines if usual ones cannot be followed	0.017	**0.676**	−0.134	−0.003
	29	Approfittare dell’occasione per coltivare i propri hobby	Taking the opportunity to cultivate hobbies	−0.143	**0.576**	−0.076	0.067
	5	Impegnarsi in qualcosa per distrarsi (ad esempio giocare o studiare)	Keeping myself busy (for example playing or studying)	−0.109	**0.534**	−0.160	−0.128
	30	Collaborare con gli altri	Collaborating with others	0.042	**0.407**	0.174	0.054
	25	Concentrarsi sulle cose veramente importanti (ad esempio la famiglia)	Focusing on things that are really important (for example family)	0.146	**0.404**	0.109	−0.055
Proactivity	36	Dare informazioni corrette, chiare e comprensibili	Giving correct, clear, and comprehensible information	−0.111	−0.082	**0.689**	0.046
	4	Informarsi tramite fonti affidabili	Looking for information from reliable sources	−0.041	−0.183	**0.622**	−0.074
	21	Aiutare e tranquillizzare chi è vicino a me	Helping and reassuring those around me	−0.038	0.205	**0.449**	0.153
	20	Chiedere informazioni in caso di dubbi sui comportamenti da tenere	In case of doubts, asking for information on appropriate behaviors	0.073	0.224	**0.405**	−0.025
	33	Comportarsi in modo sicuro (ad esempio lavando spesso le mani)	Behaving in safe ways (for example washing my hands frequently)	0.117	0.085	**0.401**	−0.285
Aversion	31	Pensare che i media e i politici stanno ingigantendo la situazione	Thinking that media and politicians are exaggerating the situation	−0.021	0.057	0.094	**0.473**
	12 (reversed)	Ricordarsi che rispettare le regole protegge la salute di tutti	Remembering that following the rules protects everybody’s health	0.001	−0.170	−0.193	**0.457**
	3	Pensare che le misure di sicurezza adottate siano inutili	Thinking that safety measures are not useful	0.113	−0.077	0.120	**0.428**
	22 (reversed)	Seguire le indicazioni degli esperti	Following advice from experts	0.004	−0.128	−0.321	**0.416**
	34	Ignorare le ordinanze del Ministero della Salute	Ignoring the regulations from the Ministry of Health	0.011	−0.043	−0.072	**0.403**

##### Power to Live With Disasters Questionnaire

The participants completed the PLDQ (Ishibashi et al., 2019) that includes 16 items to be rated on a 5-point scale (1=*not at all* and 5=*very much*). The questionnaire measures eight personality characteristics useful for coping with disasters with two items for each factor: Leadership (e.g., *In everyday life, I often take the initiative to gather people together*), Problem solving (e.g., *When I am fretting about what I should do, I compare several alternative actions*), Altruism (e.g., *When I see someone having trouble, I have to help them*), Stubbornness (e.g., *I am stubborn and always get my own way*), Etiquette (e.g., *When someone has helped me or been kind to me, I clearly convey my feelings of gratitude*), Emotional regulation (e.g., *During difficult times, I endeavor not to brood*), Self-transcendence (e.g., *I am aware that I am alive, and have a sense of responsibility in living*), and Active well-being (e.g., *In everyday life, I have habitual practices that are essential for relieving stress or giving me a change of pace*). The Italian version was adapted through back-translation.

##### Achievement Emotions Adjective List

Two months after the administration of the R-PCS, the participants completed a brief version of the Achievement Emotions Adjective List (Raccanello et al., 2021b). The respondents rated the frequency with which they had felt enjoyment or anger in the previous 2 weeks using a 5-point scale (1=*not at all* and 5=*very much*).

##### Demographics

At the end of the questionnaire, we asked for the following demographic information: year of birth, gender, course (bachelor’s degree courses, master’s degree courses, PhD, and other specialization courses), and health status with respect to the 2020 COVID-19 pandemic (tested for novel coronavirus and positive, tested for novel coronavirus and negative, not tested). In the sample, 1,476 students were 23 years of age or younger, while 1,511 students were older than 23 years. For ethical reasons, these questions were not compulsory.

### Data Analysis

The analyses were carried out using R software, Version 4.1.0 (R Core Team, 2021).

We conducted an EFA and a CFA, followed by Rasch analysis to assess the structure of the R-PCS (for a similar approach, see Raccanello et al., 2021c). We carried out the EFA on half of the sample and the CFA on the second half, after randomly splitting the initial sample into two sub-groups. To check whether the data were suitable for factor analyses, we ran the Bartlett’s test of sphericity and the Kaiser-Meyer-Olkin test (KMO; *check_factorstructure*, function parameters R package; Lüdecke et al., 2020). The first determines whether there is a significant correlation in the data while the second examines the sample adequacy. Then, we ran a parallel analysis (Horn, 1965) and an optimal coordinates analysis (Ruscio and Roche, 2012) with the scree plot (*nScree* function, nFactors R package; Raiche and Magis, 2020), and a very simple structure analysis (*vss* function, psych R package; Revelle, 2021) to identify the appropriate number of factors for the EFA (Revelle and Rocklin, 1979). We applied both EFA (*fa* function, psych R package) and CFA (*cfa* function, lavaan R package; Rosseel, 2012) for ordinal data, beginning from a polychoric correlation matrix and using maximum likelihood and an oblique promax rotation for the EFA, and the DWLS estimator for the CFA. For the EFA, we used the root-mean-square error of approximation (RMSEA) and the comparative fit index (CFI), with RMSEA ≤0.08 and CFI≥0.90 as threshold values to assess the goodness of fit; for the CFA, we also used the standardized root mean residual (SRMR) and the Tucker-Lewis index (TLI), respectively, with SRMR ≤0.08 and TLI≥0.90 (Hu and Bentler, 1999; Marsh et al., 2005). Considering that when running a CFA, the minimum ratio between the number of observations and the number of parameters should be 5:1 or more, and preferably 10:1 (Kline, 2016), and, that in our case, we had 113 estimated parameters with 1,494 participants (about 13:1), the size of our sample was appropriate. We then conducted multigroup CFA by testing separate nested CFA models, analyzing: (1) the configural invariance model, allowing all the parameters to be freely estimated; (2) the metric invariance model, requiring invariant factor loadings; and (3) the scalar invariance model, additionally requiring invariant intercepts. To compare the models, we took into account differences in CFI, RMSEA, and SRMR: Support for invariance requires a change in CFI less or equal than 0.010, a change in RMSEA less or equal than 0.015, and a change in SRMR less or equal to 0.030 for testing metric invariance and less or equal to 0.010 for testing scalar invariance (Chen, 2007).

We then verified whether the data from the whole sample fitted the Rasch model (Andrich, 1988; *PCM* function, eRm R package; Mair et al., 2021), for each dimension identified by the CFA. First, we reviewed monotonicity, to check whether the thresholds, i.e., the transition points between two different scores, were correctly ordered. To do this, we used person-item maps. Then we checked for the possible presence of local dependence between the responses to the different items for each dimension. As the next step, we conducted one CFA for each dimension of the R-PCS (for a total of four separate unidimensional CFA) to confirm their unidimensionality – one of the assumptions of the Rasch model, which must be verified; then, we calculated the standardized P-DIF statistic (Dorans and Kulick, 1986) to determine whether there was a DIF across gender and age of respondents. Moreover, we examined the reliability calculating the PSI. After all these preliminary checks, we tested the fit of the data to the Rasch model using Andersen’s likelihood ratio test (Andersen, 1973). We then examined the item performance studying infit (i.e., mean square inlier-sensitive fit) and outfit (i.e., mean square outlier-sensitive fit), considering the thresholds for rating scale surveys (Wright and Linacre, 1994). When item mean-squares are higher than 1.40, it means that the items underfit the Rasch model; when the mean-squares are lower than 0.60, it means that the items overfit the Rasch model. At the end of this series of steps, we transformed the raw scores into logit scores for use in all the following analyses.

We then examined the discriminant validity of the R-PCS (second aim) following the approach of Rönkkö and Cho (2020). We calculated the intercorrelations and the descriptive statistics between its dimensions and the factors of the PLDQ to assess the discriminant validity of the R-PCS. Correlations between 0.10 and 0.30 can be considered as small, between 0.30 and 0.50 as moderate, and higher than 0.50 as large (Cohen, 1988). We checked the eight-factor structure of the scale through a CFA. Subsequently, we investigated whether the four dimensions of the R-PCS (logit scores) predicted 2-month-after enjoyment and anger ratings, running four linear mixed models (LMM; *lmer* function, lme4 R package; Bates et al., 2015), with each separate dimension as numeric fixed effects, participants as the random effect, and emotions (enjoyment and anger) as dependent variables. Finally, we examined gender and age differences on the dimensions of the R-PCS, conducting a LMM, with gender (males and females), age (23 years of age or less and older than 23 years), and dimensions of the R-PCS as categorical fixed effects, and participants as the random effect. The logit scores of each dimension of the R-PCS were the dependent variables. We performed a type III analysis of variance table with Satterthwaite’s method. We used the Bonferroni correction for *post-hoc* tests (*emmeans* function, emmeans R package; Lenth, 2021). The level of significance was *p*<0.05.

## Results

### Structure of the R-PCS

#### Exploratory and Confirmatory Factor Analyses

**Figure 1:**
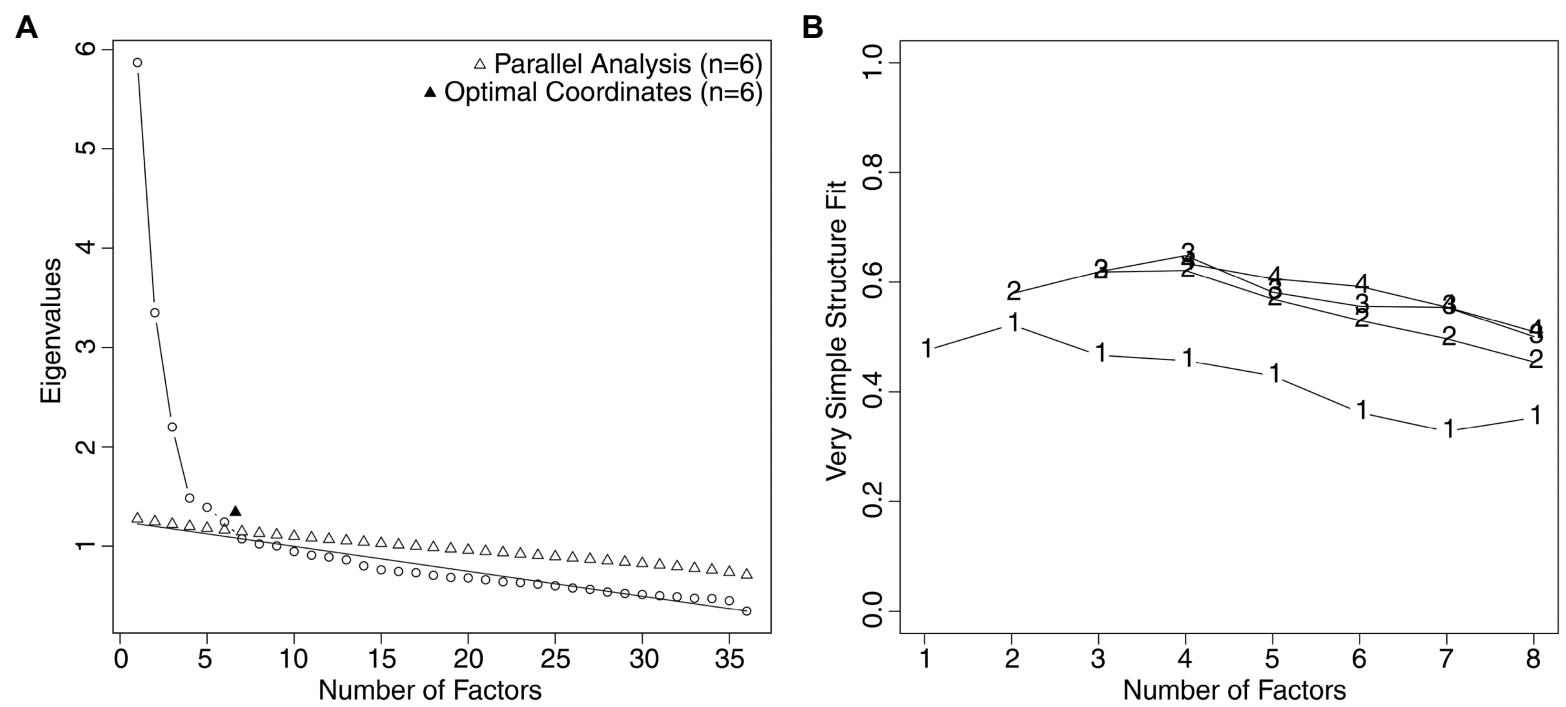
**(A)** Parallel analysis scree plot and optimal coordinates plot related to the R-PCS, suggesting that the best solution was at six factors. **(B)** The very simple structure plot, indicating that the best solution was at four factors.

The preliminary analyses conducted on half sample (*n*=1,493) indicated that the data were appropriate for factor analysis (Bartlett’s test of sphericity: *Χ*^2^(630)=11639.75, *p*<0.001; KMO=0.88). The parallel analysis and the optimal coordinates analysis suggested that the most appropriate number of factors to extract was six (see the scree plot in Figure 1A), while the analysis of the very simple structure suggested that it was four (Figure 1B).

We ran the EFA, extracting six factors. The fit indexes revealed the adequacy of the model, CFI=0.99, RMSEA=0.02. We used items with factor loadings larger than 0.40 to define each factor. Given that the fifth factor had only two items with loadings larger than 0.40, and the sixth factor had only one item with a loading larger than 0.40, and that the very simple structure analysis had suggested extracting four factors, we decided to keep only the first four factors for subsequent analyses. The factor loadings of the selected items are shown in Table 2. Note that items 12, 22, and 27 were reverse scored before the EFA.

The first dimension (which explained the 26% of the variance) included one item on helplessness (item 10), two items on delegation (item 6 and 27 – the latter was developed to assess self-reliance, but given that the score was reversed, we can interpret it in terms of its opposite strategy, i.e., delegation), and two items on submission (items 7 and 32). This dimension, we called as “Despair,” referred to the level of immobility of people who are overwhelmed by panic and lose any hope, both cognitively and emotionally. They are blocked and continue to ruminate on the emergency: They do not react, except through panic and despair, and they are convinced that there are no solutions to the problem. Overall, this factor comprises threats to the three basic needs, i.e., competence, relatedness, and autonomy.

The second dimension (which explained the 19% of the variance) included two items on accommodation (items 5 and 29) and two items on negotiation (items 25 and 26), all pertaining to challenges to the need for autonomy. In addition, it had one item related to support seeking/giving (item 30), which is also reflected in item 25 that mentions family relationships. This dimension, named as “Adjustment,” refers to the level with which people react in an adaptive and constructive way within the broad context of both individuals and activities. Overall, this factor focused on the challenges to relatedness and autonomy.

The third dimension (which explained the 19% of the variance) included one item related to problem solving (item 33) and three items related to information seeking/giving (items 4, 20, and 36). These items all concern challenges to the need for competence. Also, one item originally developed for assessing support giving (item 21) loaded on this dimension, which nevertheless referred to performing active prosocial actions to solve a problem. We termed this dimension “Proactivity,” reflecting the level to which people activate themselves to find solutions to problems, through behaviors aimed at protecting health, seeking and understanding reliable information, and helping others.

The fourth dimension (which accounted for the 17% of the variance) included two items pertaining to opposition (items 31 and 34) and other three items that had been developed, initially, for assessing problem solving (item 22, reversed), negotiation (item 12, reversed), and submission (item 3), respectively. All the items included reference to explicit opposition to rules. We called this dimension as “Aversion” as it reflected the extent to which people fail to accept the health protection rules established by competent authorities. This factor focused on the threats to the need for autonomy.

**Figure 2:**
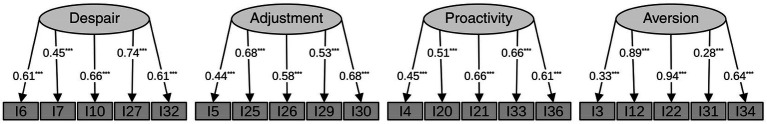
Factorial model of the R-PCS. The digits represent standardized factor loadings. ^***^p<0.001.

In Table 1, we show the four dimensions and their relationship to Zimmer-Gembeck and Skinner’s (2011) classification; in particular, their potential links to what have been described as challenges/threats and the three basic needs. Following the EFA, we performed a CFA on the other half of the sample (*n*=1,494) with four factors (Figure 2). The fit indexes, CFI=0.950, TLI=0.940, RMSEA=0.062, and SRMR=0.067, indicated that there was a relatively good fit between the hypothesized model and the observed data. Therefore, the CFA supported the idea that the R-PCS is characterized by four distinct dimensions, two related to challenges (i.e., Adjustment and Proactivity) and two related to threats (i.e., Despair and Aversion), confirming Hypothesis 1a.

Finally, to test MI, we conducted a sequence of gradually more restrictive tests to verify the configural, metric, and scalar invariance (Table 3). The results indicated that for the R-PCS the hypothesized measurement model was invariant and generalizable across both gender and age of respondents, for all the levels of invariance, corroborating Hypothesis 1b.

**Table 3:** Results of invariance analyses across gender (males, females) and age (younger than or as old as 23years, older than 23years).

Groups	Model	CFI	RMSEA	SRMR	Δ CFI	Δ RMSEA	Δ SRMR
Gender	Configural invariance	0.947	0.062	0.066	0.003	<0.001	<0.001
	Metric invariance	0.945	0.061	0.068	0.002	<0.001	0.001
	Scalar invariance	0.941	0.059	0.067	0.004	<0.002	<0.001
Grade	Configural invariance	0.947	0.063	0.068	0.003	0.001	<0.001
	Metric invariance	0.946	0.062	0.068	<0.001	0.001	<0.001
	Scalar invariance	0.945	0.058	0.068	0.001	0.004	<0.001

*N=1,494. CFI=comparative fit index; RMSEA=root-mean-square error of approximation; SRMR=standardized root-mean-square residual; Δ CFI/RMSEA/SRMR=change in CFI/RMSEA/SRMR*.

#### Rasch Analysis

**Figure 3:**
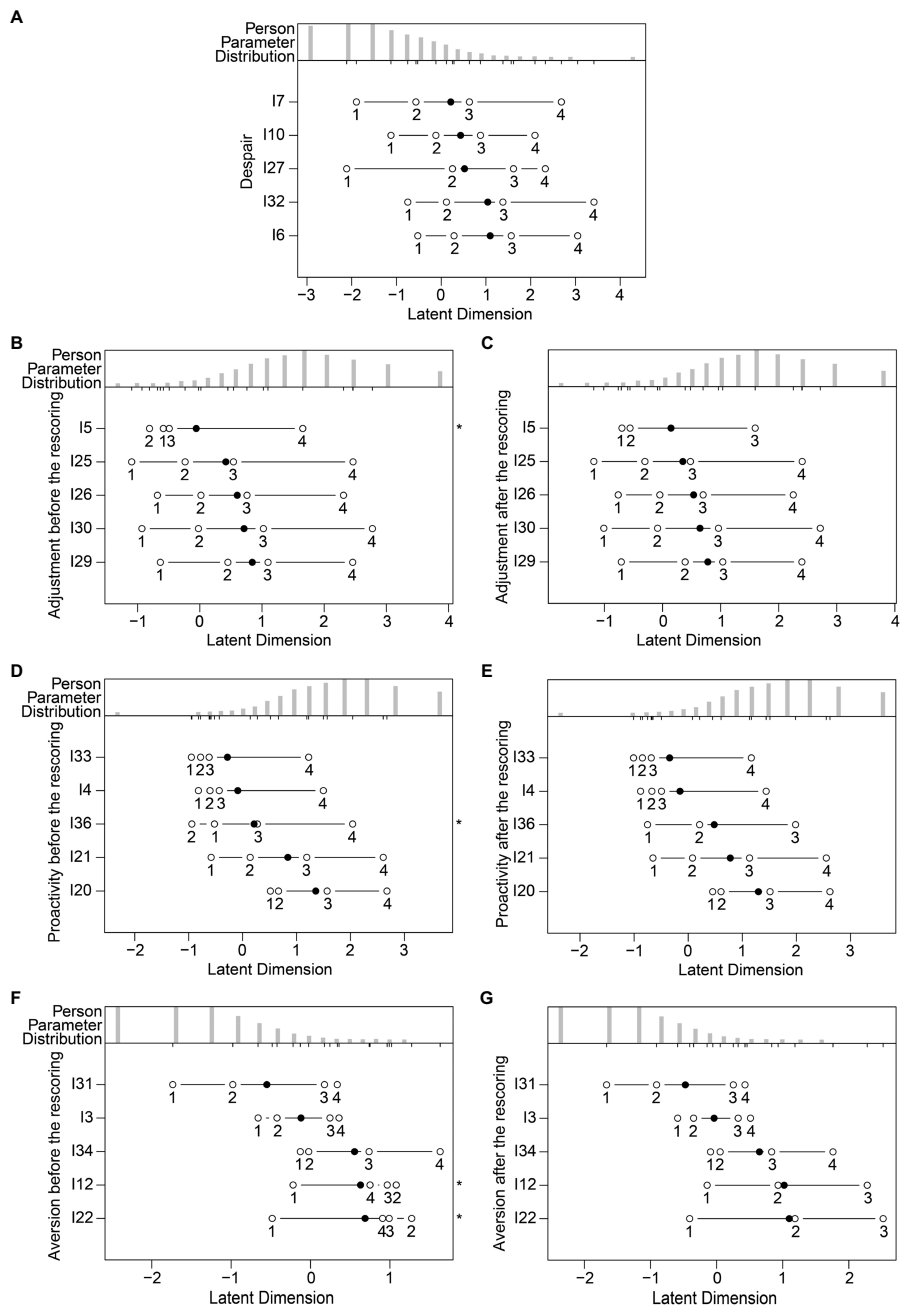
Person-item maps relating to the five items of each dimension of the R-PCS for: **(A)** Despair, without rescoring; **(B)** Adjustment, before rescoring; **(C)** Adjustment, after rescoring; **(D)** Proactivity, before rescoring; **(E)** Proactivity, after rescoring; **(F)** Aversion, before rescoring; **(G)** Aversion, after rescoring. We represented the locations of the items’ discriminatory capacities through solid circles and the thresholds through open circles. We indicated the items with non-ordered thresholds with asterisks.

We applied the Rasch analysis using the partial credit model (Masters and Wright, 1997), separately for each dimension of the R-PCS, i.e., Despair, Adjustment, Proactivity, and Aversion. First, we checked the monotonicity using person-item maps (Figure 3). A person-item map represents the relation between the location of a person’s coping strategies and the items’ discriminatory capacities. In Figure 3, the parameter related to the person (i.e., coping strategies) varies from lower scores on the left to higher scores on the right of the figures. The maps indicated that the scores of four items in the Adjustment (item 5), Proactivity (item 36), and Aversion (items 12 and 22) dimension did not have ordered thresholds (Figures 3B,D,F). Thus, we rescored them because they violated the monotonicity assumptions (we specify that we had reversed the scores of items 12 and 22 before recoding them). For items 5 and 36, the response scale changed from 1, 2, 3, 4, 5 to 1, 1, 2, 3, 4; for items 12 and 22, it changed from 1, 2, 3, 4, 5 to 1, 2, 3, 4, 4. This resulted in the items in the three factors, i.e., Adjustment, Proactivity, and Aversion, meeting the monotonicity requirements (Figures 3C,E,G). We then examined the correlations between the item residuals (Despair: −0.36<*r*<0.10; Adjustment: −0.39<*r*<−0.14; Proactivity: −0.43<*r*<−0.01; Aversion: −0.45<*r*<0.12), which were never larger than 0.30; therefore, there was no evidence of local dependence. The CFA conducted separately for each of the four dimensions indicated that each of them was unidimensional (Despair: CFI=0.997, TLI=0.989, RMSEA=0.057, and SRMR=0.026; Adjustment: CFI=0.981, TLI=0.953, RMSEA=0.086, and SRMR=0.044; Proactivity: CFI=0.998, TLI=0.994, RMSEA=0.029, and SRMR=0.016; Aversion: CFI=0.981, TLI=0.952, RMSEA=0.076, and SRMR=0.051). Then, we calculated the standardized P-DIF statistic separately for gender (Despair: −0.071<DIF<0.086; Adjustment: −0.026<DIF<0.031; Proactivity: −0.013<DIF<0.001; Aversion: −0.079<DIF<0.049) and age (Despair: −0.016<DIF<0.016; Adjustment: −0.009<DIF<0.003; Proactivity: −0.004<DIF<0.003; Aversion: −0.015<DIF<0.032). Given that they fell between −0.10 and 0.10, we can say that each dimension functioned similarly for males and females and for younger and older participants. Concerning reliability, all the PSI were adequate (Despair: 0.71; Adjustment: 0.71; Proactivity: 0.70; Aversion: 0.70).

At this point, we carried out four separate Andersen’s likelihood ratio tests (Despair: *Χ*^2^(19)=23.000, *p*=0.237; Adjustment: *Χ*^2^(18)=12.800, *p*=0.802; Proactivity: *Χ*^2^(18)=21.800, *p*=0.242; Aversion: *Χ*^2^(17)=12.394, *p*=0.776), which showed that for each dimension the data fit the Rasch model. Also, the infit and outfit mean square statistics for each item of each dimension (Table 4) confirmed that the data were predicted by the model (all the values fell between 0.60 and 1.40). Subsequently, for each dimension and for each participant, we summed the raw scores of the five items, and we obtained a global score. Finally, we transformed the raw scores into an interval logit scale (Masters and Wright, 1997), as shown in the conversion table (Table 5). To increase the usability of the scale, the logit scores were scaled from 1 to 10 (considering that four items, i.e., 5, 12, 22, and 36, were rescored).

**Table 4:** Infit and outfit mean square statistics (MSQ) of each item of each dimension of the R-PCS.

Factor name	Item number	Infit-MSQ	Outfit-MSQ
Despair	6	0.67	0.61
	27 (reversed)	0.93	0.93
	7	0.80	0.82
	32	0.87	0.89
	10	0.90	0.90
Adjustment	26	0.95	0.95
	29	0.72	0.75
	5	0.79	0.80
	30	0.83	0.86
	25	0.84	0.84
Proactivity	36	0.84	0.90
	4	0.79	0.81
	21	0.80	0.80
	20	0.81	0.82
	33	0.84	0.88
Aversion	31	0.79	0.77
	12 (reversed)	0.82	0.79
	3	0.81	0.78
	22 (reversed)	0.87	0.86
	34	0.91	0.95

**Table 5:** Conversion table from raw scores of the items of the R-PCS to logit scores, separately for each dimension.

Logit scores				
Raw scores	Despair	Adjustment	Proactivity	Aversion
5	1.000	1.000	1.000	1.000
6	1.812	1.737	1.637	1.969
7	2.560	2.400	2.253	2.855
8	3.043	2.925	2.755	3.401
9	3.414	3.234	3.267	3.807
10	3.725	3.658	3.576	4.139
11	4.000	3.958	3.861	4.429
12	4.254	4.239	4.138	4.695
13	4.494	4.511	4.414	4.947
14	4.729	4.781	4.697	5.197
15	4.963	5.058	4.990	5.453
16	5.200	5.346	5.293	5.722
17	5.442	5.654	5.614	6.016
18	5.695	5.989	5.957	6.348
19	5.963	6.363	6.334	6.735
20	6.253	6.791	6.760	7.377
21	6.579	7.300	7.266	8.089
22	6.962	7.947	7.916	8.956
23	8.200	8.933	8.916	10.000
24	9.046	10.000	10.000	–
25	10.000	–	–	–

**Figure 4:**
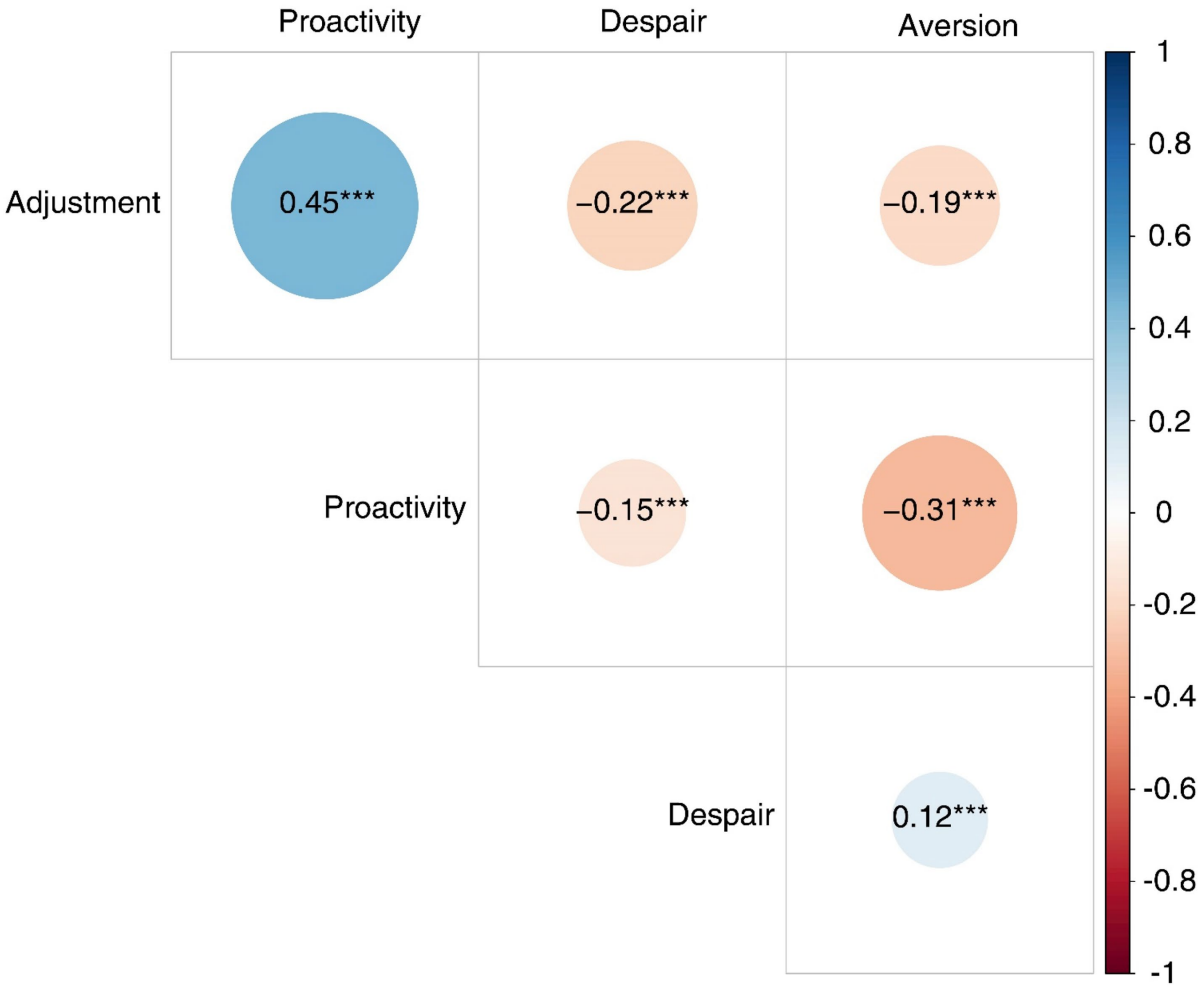
Plot of the intercorrelations between the four dimensions of the R-PCS (logit scores).

The intercorrelations and the descriptive statistics of the four dimensions of the R-PCS are shown in Table 6. The McDonald’s omega reliability indexes were 0.79, 0.72, 0.73, and 0.70, respectively, for Despair, Adjustment, Proactivity, and Aversion, suggesting that the scale had an acceptable reliability. In Figure 4, we plot the intercorrelations between the four dimensions of the R-PCS (logit scores), showing that the four-factor solution is characterized in terms of two categories, adaptive and maladaptive – i.e., the two adaptive dimensions (Adjustment and Proactivity) correlated negatively with the two maladaptive dimensions (Despair and Aversion).

**Table 6:** Intercorrelations and descriptive statistics (means, M; standard deviations, SD; 95% confidence intervals, CI) for the four dimensions of the R-PCS (logit scores) and the eight factors of the power to live with disasters questionnaire (PLDQ).

S.no.	Variable	1	2	3	4	5	6	7	8	9	10	11	12
1.	R-PCS – Despair	–											
2.	R-PCS – Adjustment	−0.22^**^	–										
3.	R-PCS – Proactivity	−0.15^**^	0.45^**^	–									
4.	R-PCS – Aversion	0.12^**^	−0.19^**^	−0.31^**^	–								
5.	PLDQ – Leadership	−0.08^**^	0.33^**^	0.32^**^	−0.13^**^	–							
6.	PLDQ – Problem solving	−0.18^**^	0.35^**^	0.35^**^	−0.13^**^	0.32^**^	–						
7.	PLDQ – Altruism	−0.03	0.31^**^	0.31^**^	−0.14^**^	0.40^**^	0.29^**^	–					
8.	PLDQ – Stubbornness	0.01	0.03	0.04^*^	0.12^**^	0.06^*^	0.11^**^	0.03	–				
9.	PLDQ – Etiquette	−0.14^**^	0.37^**^	0.34^**^	−0.15^**^	0.49^**^	0.28^**^	0.40^**^	−0.02	–			
10.	PLDQ – Emotional regulation	−0.37^**^	0.38^**^	0.30^**^	−0.12^**^	0.21^**^	0.38^**^	0.16^**^	−0.04^*^	0.26^**^	–		
11.	PLDQ – Self-transcendence	−0.12^**^	0.42^**^	0.39^**^	−0.22^**^	0.34^**^	0.34^**^	0.38^**^	−0.01	0.37^**^	0.33^**^	–	
12.	PLDQ – Active well-being	−0.13^**^	0.43^**^	0.32^**^	−0.05^*^	0.33^**^	0.42^**^	0.22^**^	0.08^**^	0.32^**^	0.35^**^	0.41^**^	–
	*M*	3.13	6.47	6.84	2.98	3.21	3.52	4.22	2.67	3.54	3.30	3.95	3.42
	*SD*	1.17	1.38	1.47	1.21	0.83	0.72	0.63	0.82	0.82	0.82	0.74	0.81
	*95% CI*	[3.09, 3.17]	[6.42, 6.52]	[6.79, 6.89]	[2.94, 3.03]	[3.21, 3.28]	[3.49, 3.54]	[4.19, 4.24]	[2.64, 2.70]	[3.51, 3.57]	[3.26, 3.32]	[3.92, 3.97]	[3.39, 3.45]

* *p<0.05*;

*p<0.01;

and

** *p<0.001*.

### Validity of the R-PCS

#### Discriminant Validity

To investigate discriminant validity, we examined the latent correlations based on the results of the CFA and their confidence intervals (CI) using a significance level of 5%, since the CFA model considers the measurement error (Table 3). We took into account the CI_CFA_ checking if the upper limit of the CI for each latent correlation was lower than 0.80. In most of the cases the upper limits were below the cut-off, ranging from 0.27 to 0.80, confirming the discriminant validity for the measures (Rönkkö and Cho, 2020). There was a significant, positive, and moderate correlation between the two adaptive dimensions (Adjustment and Proactivity), and a positive and small correlation between the two maladaptive dimensions (Despair and Aversion). The adaptive dimensions negatively correlated with the maladaptive dimensions, and in most of the cases the correlations were small (Table 6). Therefore, our data revealed that the four dimensions were separable, confirming Hypothesis 2a.

#### Criterion Validity

We conducted a preliminary CFA to test the factorial structure of the PLDQ. The model with eight factors was adequate, CFI=0.974, TLI=0.958, RMSEA=0.052, and SRMR=0.052. We then calculated the intercorrelations between the four dimensions of the R-PCS and the eight factors of the PLDQ (Table 6).

As regards the two adaptive dimensions, there were significant, positive, and moderate correlations between Adjustment and Proactivity on the one hand and most of the factors of the PLDQ on the other hand, with some exceptions (i.e., the correlation between Adjustment and Stubbornness was not significant; the one between Proactivity and Stubbornness was small). Concerning the two maladaptive dimensions, there were significant, negative, and small correlations between Despair and Aversion on the one hand and most of the factors of the PLDQ on the other hand, with some exceptions. The correlations between Despair and Altruism, and between Despair and Stubbornness, were not significant; the one between Despair and Emotional regulation was moderate; and the one between Aversion and Stubbornness was positive. Hence, the data indicated the criterion validity of the R-PCS, supporting Hypothesis 2b.

#### Predictive Validity

Adjustment was related positively to enjoyment, *β*=0.049, *p*=0.044, and negatively to anger, *β*=−0.050, *p*=0.043. Proactivity was positively related to enjoyment, *β*=0.044, *p*=0.050. Both Despair, *β*=0.150, *p*<0.001, and Aversion, *β*=0.124, *p*<0.001, were positively related to anger. Therefore, our data supported Hypothesis 2c.

### Gender and Age Differences

The LMM revealed a significant effect of the R-PCS dimensions, *F*(3, 11,948)=4718.478, *p*<0.001, *η^2^_p_*=0.83. The *post-hoc* tests indicated that the scores were higher for Adjustment (*M*=6.47, *SD*=1.38, 95% CI [6.42, 6.52]) and Proactivity (*M*=6.84, *SD*=1.47, 95% CI [6.79, 6.89]) compared to Despair (*M*=3.13, *SD*=1.17, 95% CI [3.09, 3.17]; Adjustment vs. Despair, *z*=80.15, *p*<0.001; Proactivity vs. Despair, *z*=90.12, *p*<0.001) and Aversion (*M*=2.98, *SD*=1.21, 95% CI [2.94, 3.03]; Adjustment vs. Aversion, *z*=77.51, *p*<0.001; Proactivity vs. Aversion, *z*=87.48, *p*<0.001).

**Figure 5:**
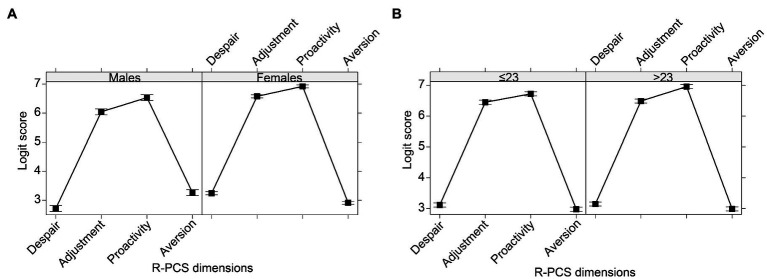
R-PCS logit scores of the four dimensions, according to **(A)** gender and **(B)** age of respondents. The bars represent the 95% CI.

Also gender, *F*(1, 11,948)=87.303, *p*<0.001, $\eta_{p}^{2}$=0.03, and age, *F*(1, 11,948)=6.770, *p*=0.009, $\eta_{p}^{2}$=0.01, had significant effects, in turn moderated by two significant two-way interactions, gender X R-PCS dimensions, *F*(3, 11,948)=51.616, *p*<0.001, $\eta_{p}^{2}$=0.05 (Figure 5A), and age X R-PCS dimensions, *F*(3, 11,948)=3.841, *p*=0.009, $\eta_{p}^{2}$=0.01 (Figure 5B). Examining the *post-hoc* tests, we found that males had lower scores than females for Despair (males: *M*=2.72, *SD*=1.12, 95% CI [2.63, 2.80]; females: *M*=3.24, *SD*=1.16, 95% CI [3.19, 3.29]; *z*=−9.00, *p*<0.001), Adjustment (males: *M*=6.04, *SD*=1.34, 95% CI [5.94, 6.15]; females: *M*=6.58, *SD*=1.37, 95% CI [6.52, 6.63]; *z*=−9.07, *p*<0.001), and Proactivity (males: *M*=6.54, *SD*=1.45, 95% CI [6.43, 6.66]; females: *M*=6.92, *SD*=1.47, 95% CI [6.86, 6.98]; *z*=−6.58, *p*<0.001), while they had higher scores for Aversion (males: *M*=3.26, *SD*=1.20, 95% CI [3.17, 3.36]; females: *M*=2.91, *SD*=1.20, 95% CI [2.86, 2.96]; *z*=5.96, *p*<0.001). Concerning age, only for Proactivity the scores were lower, *z*=−4.19, *p*<0.001, for younger (*M*=6.73, *SD*=1.48, 95% CI [6.65, 6.80]) compared to older students (*M*=6.95, *SD*=1.46, 95% CI [6.88, 7.02]).

## Discussion

We developed a brief, reliable, and valid scale to assess adaptive and maladaptive coping strategies related to pandemics and epidemics, based on Zimmer-Gembeck and Skinner’s (2011) classification. Importantly, by fitting a Rasch model to the data collected for this project, we have created a scale in which the item scale values are independent of the sample of people who completed the items during scale development. This means that the items will have the same scale properties in any sample in which it is used and an asset for researchers working in the area.

The relevance of this paper can be appreciated considering methodological (first and second aim), theoretical (third aim), and applied perspectives.

Our first aim was to examine the structure of the R-PCS. Through a dual approach combining an EFA and a CFA, we identified the factorial structure of the R-PCS, including four first-order dimensions, namely Despair, Adjustment, Proactivity, and Aversion. Confirming Hypothesis 1a, we found two dimensions focused on challenges and two dimensions focused on threats. Based on the psychological literature (Zimmer-Gembeck and Skinner, 2011), we could speculate that the first two were adaptive while the other two were maladaptive. Adjustment included items pertaining to challenges to relatedness and autonomy, while Proactivity items concerning challenges to competence. Despair comprised items referred to threats covering all the three needs, i.e., competence, relatedness, and autonomy, while Aversion was specifically focused on items on threats to individuals’ autonomy. We could consider that the overall capacity to overcome stressful events can result from the combination of the different dimensions. Therefore, future studies could explore individuals’ profiles concerning how they react to traumatic events, such as the COVID-19 pandemic, and other similar stressful events. They could identify which combinations of different levels of endorsement of Despair, Adjustment, Proactivity, and Aversion are associated with individuals’ emotional reactions or other indicators of mental disturbance or positive psychological functioning.

We also tested the measurement invariance of the R-PCS, which was invariant at the configural, metric, and scalar levels both across gender and across age. We then applied the Rasch model, transforming the scores of each dimension into interval level measures, with all the advantages related to the principles of the fundamental measurement (Rasch, 1960; Andrich, 1988; Bond and Fox, 2007; Burro, 2016).

Concerning the second aim, our findings showed the discriminant validity of the R-PCS, revealing that the four identified dimensions were independent and separable, confirming Hypothesis 2a. Moreover, the analysis of the correlations with the PLDQ showed good criterion validity, also supporting Hypothesis 2b. Concerning predictive validity, we examined the relationships between the four dimensions of the R-PCS and two emotions measured after 2 months, and our data confirmed Hypothesis 2c. Adjustment and Proactivity appeared adaptive, being both positively related to enjoyment while Adjustment was also negatively related to anger; Despair and Aversion seemed maladaptive, i.e., positively related to anger. Therefore, our findings supported the theoretical assumptions (Zimmer-Gembeck and Skinner, 2011) of the adaptive nature of coping strategies focused on challenges (Adjustment and Proactivity), and the maladaptive nature of those focused on threats (Despair and Aversion).

Among our sample of Italian university students (third aim), the scores of the two dimensions focused on challenges, i.e., Adjustment and Proactivity, were higher than the scores of the two dimensions focused on threats, i.e., Despair and Aversion. These findings suggest that, during the first wave of the COVID-19 pandemic, the students still perceived that they had adaptive resources, which enabled them to face such a stressful event. Future research could investigate how and whether these resources changed in the face of the continuous and persistent emergency phase of the pandemic. Moreover, females were characterized by higher scores on Despair, Adjustment, and Proactivity than males and vice versa for Aversion. The findings concerning Despair and Aversion are in line with the previous literature on the prevalence of internalizing behaviors for females and of externalizing behaviors for males, especially since adolescence (Rosenfield, 2000). Finally, Proactivity increased for older students. In any case, the effect sizes of all these differences were quite low. Therefore, these differences could be explored further, examining possible links with other constructs, and specifically other differential adjustments to the pandemic over time.

At the applied level, being able to measure pandemic-related coping strategies is of basic relevance for subsequent interventions. The literature has documented that, at least for university students, being able to verbalize emotions and having access to a range of coping strategies is positively linked to their quality of life during the pandemic (Panayiotou et al., 2021). Therefore, during and after disasters it is a priority to have instruments to detect how people are reacting and to identify in which areas they have difficulties. For example, the R-PCS could be used to monitor university students’ coping strategies during the different phases of a pandemic to inform policy decisions. In addition, it could be applied before and after interventions aimed at supporting adults in coping with the emotional challenges of a pandemic, to assess the efficacy of the interventions. Moreover, it could be used with patients to help in prescribing appropriate individualized interventions aimed at fostering emotional competence.

This study suffers from some limitations. One limitation is that the final version of the R-PCS did not include all the coping strategies of Zimmer-Gembeck and Skinner’s (2011) classification, because of the mediocre factorial loading of the corresponding items. Specifically, it did not comprise escape and social isolation. For both coping strategies, we could speculate that this could be linked to the contents of the items themselves. For example, the escape-related item *Pretending that there is no emergency* could refer more to a psychiatric symptom than to a proper coping strategy. In addition, the social-isolation item *Being selfish* could be particularly affected by social desirability biases and therefore being associated with a different pattern of responses compared to the other items. In future studies, we could reformulate the items relating to the excluded coping strategies to expand the scale. It is also worth noting that, in the psychological literature, there are several classifications of coping, and therefore we do not claim that our scale captures every type of coping. Moreover, the responses to the whole questionnaire could have suffered from a social desirability bias. One way to deal with this issue is to assure the confidential nature of the data, fostering people’s tendency to trust the researchers (Pekrun and Bühner, 2014). Another limitation relates to the gender imbalance in our sample, with the majority of participants being female. It is worth noting that such imbalance is consistent with the percentages of females (63.9%) attending the University of Verona during the academic year 2019–2020. Moreover, the unbalanced composition of our sample could also be due to the fact that females were more prone to spend some time in an activity that was seen as having prosocial aims, i.e., completing a survey to increase knowledge on the emotional consequences of the pandemic. We could speculate that this is in line with gender stereotype according to which girls engage in more prosocial behaviors (Hastings et al., 2015). Finally, the recruitment of the sample could have been biased by self-selection effects, and we could not investigate the reasons underlying the decision not to respond to the survey; a critical aspect of most of the research on these topics conducted during the 2020 pandemic. In addition, we specify that the generality of our findings can be extended to students of the same age of similar socio-cultural contexts who are living the emergency phase of a disaster with characteristics similar to the ongoing pandemic. On the one hand, we unfortunately note that, currently, the COVID-19 pandemic is still on course, and therefore, there are potentially many students who are in a situation similar to the one that characterized our sample when they participated to our survey. Moreover, the R-PCS could be used also in post-pandemic assessment with samples with similar characteristics. On the other hand, future research could replicate our findings with samples varying for other characteristics, to favor the robust advancement of the scientific knowledge about how to cope in front of disasters. We have no reason to believe that the results depend on other characteristics of the participants, materials, or context (Simons et al., 2017). Notwithstanding these limitations, the current study offers a new instrument to assess pandemic-related coping strategies, the R-PCS, whose psychometric properties benefit from the strengths of the Rasch model. Even if the scale has been developed during a disaster, such as the COVID-19 pandemic, it can be used to measure coping strategies in all the phases of the disaster management cycle, i.e., before, during, and after a pandemic or an epidemic. Always considering Zimmer-Gembeck and Skinner’s (2011) classification as the theoretical framework, in the future the scale can also be adapted to other disasters and for different age groups.

## Data Availability Statement

The raw data supporting the conclusions of this article will be made available by the authors, without undue reservation.

## Ethics Statement

The studies involving human participants were reviewed and approved by the Ethical Committee of the Department of Human Sciences of the University of Verona (Italy). The participants provided their written informed consent to participate in this study.

## Author Contributions

RB, DR, and GV contributed to conception, design of the study, and organized the database. RB performed the statistical analysis. RB and DR wrote the first draft of the manuscript. RB, DR, GV, VB, ER, and RH wrote sections of the manuscript. All authors contributed to manuscript revision, and read and approved the submitted version.

## Funding

This work was supported by the Fondazione Cariverona, Bando Ricerca Scientifica 2017, Italy.

## Conflict of Interest

Author Rob Hall is employed by Environmetrics Pty Ltd.

The remaining authors declare that the research was conducted in the absence of any commercial or financial relationships that could be construed as a potential conflict of interest.

## Publisher’s Note

All claims expressed in this article are solely those of the authors and do not necessarily represent those of their affiliated organizations, or those of the publisher, the editors and the reviewers. Any product that may be evaluated in this article, or claim that may be made by its manufacturer, is not guaranteed or endorsed by the publisher.
